# Differences in Chemosensitivity between Primary and Metastatic Tumors in Colorectal Cancer

**DOI:** 10.1371/journal.pone.0073215

**Published:** 2013-08-28

**Authors:** Katsushi Takebayashi, Eiji Mekata, Hiromichi Sonoda, Tomoharu Shimizu, Hisanori Shiomi, Shigeyuki Naka, Yoshihiro Endo, Tohru Tani

**Affiliations:** Department of Surgery, Shiga University of Medical Science, Otsu, Shiga, Japan; Virginia Commonwealth University, United States of America

## Abstract

**Purpose:**

We retrospectively evaluated the in vitro chemosensitivity of primary site and metastatic site tumors in colorectal cancer.

**Methods:**

Various resected tumor samples (33 from lymph nodes, 42 from liver, six from lung, and 68 primary tumors) were assessed via a collagen gel droplet-embedded culture drug sensitivity test to determine chemosensitivity to a single agent or a combination of agents.

**Results:**

Sensitivity to combination chemotherapy was significantly higher than that of monotherapy in the primary site group, lymph node group, and liver group. There was significant difference between chemosensitivity of primary site and that of liver metastasis in each agent (5-FU, p<0.001; SN38, p = 0.045; 5-FU/SN38, p<0.001; OHP, p = 0.037; 5-FU/OHP, p = 0.045).

**Conclusions:**

Tumors showed greater in vitro chemosensitivity to combination therapy when compared with monotherapy. Further, tumors that had metastasized to the liver were more resistant to chemotherapy when compared with matched primary tumors.

## Introduction

The collagen gel droplet-embedded culture drug sensitivity test (CD-DST) is an in vitro anticancer drug sensitivity test [1]–[6]. Recent studies have reported that CD-DST can provide useful therapeutic information for patients with gastric cancer, lung cancer, colorectal cancer or pancreatic cancer [7]–[12]. Furthermore, CD-DST can assess sensitivity to newer agents. We previously described the clinical potential of CD-DST in patients with colorectal cancer (CRC) [12], [13] in terms of identifying chemoresistant and chemosensitive tumors [12], [13]. CRC is one of the leading causes of death worldwide and continues to increase in incidence. More than half of patients who are initially diagnosed with localized disease ultimately develop stage IV CRC [14]. In most instances, synchronous metastases are not resectable. The main treatment for metastatic CRC is chemotherapy, and recent advances in systemic chemotherapy have resulted in improved outcomes for these patients. However, the clinical response to chemotherapy differs when comparing primary versus metastatic tumors (e.g., in the lymph nodes, liver, or lungs), and patients with chemoresistant tumors might benefit from other types of treatment strategies [15]–[19].

The aim of the present study was to evaluate the difference in in vitro chemosensitivity in primary versus metastatic tumors in patients with CRC. Our study demonstrated the difference of in vitro chemosensitivity in primary and metastatic colorectal cancer.

## Patients and Methods

### Patients

The CD-DST was performed in 940 tissues (588 primary tumors; 78 tumors in lymph nodes, 124 tumors in in liver, and 27 tumors in lung) taken from patients with CRC between August 1999 and December 2011 at Shiga University of Medical Science in Japan. Tumors with >70% growth rate (33 from lymph nodes, 42 from liver, six from lung, and 68 primary tumors) were selected for subsequent analysis. A total of 68 primary tumors were assessed, each of which was resected from the same patient in which the metastatic tumors were resected. Comparative chemosensitivities to various single or combination treatments were compared among the different tumor sites.

All patients were younger than 85 years and had untreated evaluable metastatic sites that were diagnosed by computed tomography (CT), 18F-fluorodeoxyglucose positron emission tomography-computed tomography (FDG-PET-CT), and/or diffusion-weighted magnetic resonance imaging (DW-MRI). The primary tumors and metastatic tumors were surgically resected, and all tissue samples were investigated by CD-DST to evaluate their chemosensitivities. All samples were histologically confirmed as colorectal adenocarcinoma.

This study conformed to the Clinical Research Guidelines of Shiga University of Medical Science, and was approved by the research ethics committee at Shiga University of Medical Science. We obtained written informed consent to participate in this study from all patients.

### Collagen Gel Droplet-embedded Culture Drug Sensitivity Test (CD-DST)

The CD-DST was performed using tumor tissue, as previously described by Kobayashi et al. [4], [5]. Briefly, surgically resected specimens were digested in dispersion collagenase enzyme, and the dispersed cancer cells were incubated in a collagen gel-coated flask. Then, the viable cells adhering to the collagen gel-layer were collected and were added to reconstructed type 1 collagen solution (Cell matrix Type CD™, Kurabo, Osaka, Japan). Three drops of these mixtures were placed in each well of a six-well plate, and then 5-fluorouracil (5-FU) (1.0 µg/ml), irinotecan (SN38) (0.03 µg/ml), oxaliplatin (OHP) (0.5 µg/ml), 5-FU/SN38 (1.0 µg/ml, 0.03 µg/ml), or 5-FU/OHP (1.0 µg/ml, 0.5 µg/ml) was added to each well. Plates were incubated for 24 hours. After removal of the medium containing the anticancer drug(s), each well was incubated with PCM-2 medium (Kurabo) for 7 days. The in vitro chemosensitivity effect of each agent was expressed as a ratio of the total colony volume (T) of the treated cells to that of the untreated cells (C). A sample with a ratio of T to C of 60% or less was regarded as chemosensitive [6], [12].

### Statistical Analysis

Statistical analyses for baseline characteristics were performed using the JMP software program version 9 (SAS Institute Inc. Cary, NC, USA). The Chi-square test was used to analyze data. The Student’s t test was used to compare CD-DST data. Analyses were conducted using Excel (Microsoft, Redmond, WA) and Statcel2 (OMS Publisher, Saitama, Japan) software. A p value of less than 0.05 was considered to indicate statistical significance.

## Results

### Patient Characteristics

Patient characteristics are shown in Table 1. Baseline characteristics were balanced in each group. In the lymph node metastasis group, primary tumors were in the colon in 19 patients and in the rectum in 14 patients. In the liver metastasis group, primary tumors were in the colon in 24 patients and in the rectum in 18 patients. In the lung metastasis group, primary tumors were in the colon in two patients and in the rectum in four patients. A total of 68 primary tumors were assessed, each of which was resected from the same patient in which the metastatic tumors were resected.

**Table 1:** Characteristics of patients with metastatic colorectal cancer.

	Lymph node (n = 33)	Liver (n = 42)	Lung (n = 6)	Primary site (n = 68)	P value
Age (median) (years)	67.64 (52–85)	65.57 (52–85)	68.26 (36–81)	66.62 (36–85)	0.4259
Gender (male/female)	20/13	22/20	4/2	39/29	0.6158
Primary tumor site (colon/rectum)	19/14	24/18	2/4	37/31	0.5055

### Chemosensitivity to Single Agent Chemotherapy Versus Combination Chemotherapy

Sensitivities to chemotherapy are summarized in Table 2. Sensitivity was greater to 5-FU/SN38 therapy than to 5-FU therapy in the following tumor types: primary tumors (p<0.001), lymph node tumors (p<0.001), liver tumors (p<0.001). Sensitivity was greater to 5-FU/OHP therapy than to 5-FU therapy in the following tumor types: primary tumors (p = 0.0014), lymph node tumors (p<0.001), liver tumors (p = 0.03).

**Table 2:** Evaluation of combination agents in CD-DST.

T/C (%)	SN38	5-FU	5-FU/SN38	OHP	5-FU	5-FU/OHP
		p<0.001*			p = 0.0014*	
Primary site (n = 68)	67.47 (0–100)	72.9 (0–100)	56.78 (0–100)	63.1 (0–100)	72.9 (0–100)	58.7 (0–100)
		p<0.001*			p<0.001*	
Lymph node (n = 33)	69.76 (32–100)	75.75 (37–100)	57.23 (0–94.9)	56.39 (0–100)	75.75 (37–100)	49.32 (0–100)
		p<0.001*			p = 0.03*	
Liver (n = 42)	73.26 (31–100)	83.28 (41–100)	65.36 (0–100)	81.5 (0–100)	83.28 (41–100)	66.77 (0–100)
						
Lung (n = 6)	64.49 (54–77)	77.05 (61–94)	58.99 (42–70)	79.02 (65–93)	77.05 (61–94)	66.69 (36–83)

The in vitro sensitivity was expressed as the T/C ratio, in which T is the total volume of living cancer cells in the treated group, and C is the total volume of living cancer cells in the control group. Positive, T/C <60%; negative, T/C ≥ 60%.

5-FU, 5-fluorouracil; SN38, the active metabolite of irinotecan; OHP, the active metabolite of oxaliplatin.

* statistically significant.

There was no significant difference in chemosensitivity to single agent chemotherapy versus that to combination chemotherapy in lung tumors.

### Chemosensitivity of Primary Versus Metastatic Tumors

There was no significant difference in chemosensitivity when comparing lymph node tumors and matched primary tumors (Table 3). Chemosensitivity was significantly lower in liver tumors than that of matched primary tumors (5-FU, p<0.001; SN38, p = 0.045; 5-FU/SN38, p<0.001; OHP, p = 0.037; 5-FU/OHP; p = 0.045) (Table 4). There was no significant difference in chemosensitivity when comparing lung tumors and matched primary tumors (Table 5).

**Table 3:** Comparison of chemosensitivity between primary site and lymph node metastasis in CD-DST.

Agent	T/C (%) in primary site (n = 33)	T/C (%) in lymph node (n = 33)	p value
5-FU	74.26 (0–100)	75.75 (37–100)	0.37
SN38	69.39 (0–100)	69.76 (32–100)	0.47
5-FU/SN38	53.42 (0–100)	57.23 (0–94.9)	0.48
OHP	58.21 (0–100)	56.39 (0–100)	0.41
5-FU/OHP	56.39 (0–100)	49.32 (0–100)	0.22

5-FU, 5-fluorouracil; SN38, the active metabolite of irinotecan; OHP, the active metabolite of oxaliplatin.

**Table 4:** Comparison of chemosensitivity between primary site and liver metastasis in CD-DST.

Agent	T/C (%) in primary site (n = 42)	T/C (%) in liver metastasis (n = 42)	p value
5-FU	71.99 (0–100)	83.28 (41–100)	<0.001*
SN38	66.37 (0–100)	73.26 (31–100)	0.045*
5-FU/SN38	56.31 (0–100)	67.34 (28–100)	<0.001*
OHP	62.35 (0–100)	76.49 (0–79.3)	0.037*
5-FU/OHP	58.17 (0–100)	67.46 (0–100)	0.045*

5-FU, 5-fluorouracil; SN38, the active metabolite of irinotecan; OHP, the active metabolite of oxaliplatin.

There was no significant difference in chemosensitivity when comparing the liver metastasis and the primary tumor.

* statistical significance.

**Table 5:** Comparison of chemosensitivity between primary site and lung metastasis in CD-DST.

Agent	T/C (%) in primary site (n = 6)	T/C (%) in lung metastasis (n = 6)	p value
5-FU	72.06 (0–100)	77.05 (61–94)	0.38
SN38	61.07 (36.5–100)	64.49 (54–77)	0.37
5-FU/SN38	53.67 (0–98)	58.99 (42–72)	0.48
OHP	70.94 (60–100)	70.38(65–100)	0.36
5-FU/OHP	56.68 (25.6–72.6)	66.69 (36–83)	0.17

5-FU, 5-fluorouracil; SN38, the active metabolite of irinotecan; OHP, the active metabolite of oxaliplatin.

There was no significant difference in chemosensitivity when comparing the lung metastasis and the primary tumor.

## Discussion

The present study demonstrated the difference of in vitro chemosensitivity of colorectal primary and metastatic tumors, and showed that combination chemotherapy was more than effective than monotherapy.

The results of the CD-DST in vitro anticancer drug sensitivity test [1]–[6] correlate well with patient outcomes; patients treated with regimens that were deemed “sensitive” according the CD-DST assay had better outcomes when compared with those patients who were treated with regimens that were deemed “insensitive” [12], [13]. Since patients whose tumors are deemed chemoresistant based on CD-DST testing may not benefit from such therapy, other therapeutic approaches should be considered for those patients [15]–[19].

Management of metastatic CRC may consist of medical treatments (e.g., conventional systemic chemotherapy, molecularly targeted agents) and/or surgery. While 5-FU, SN38, and OHP are the standards of care for the treatment of CRC, some patients may be resistant to these therapies. Therefore, it would be beneficial to proactively identify which agents are expected to be effective versus those expected to be ineffective. George et al. reported that 93% of patients with synchronous stage IV CRC who received chemotherapy did not require palliative surgery for the primary tumor [20]. Primary tumor resection is performed if the patient is symptomatic and is therefore considered on an individual basis. Mentha et al. described a new strategy for the management of liver metastasis [21] in which initial control and downstaging of liver metastasis before primary tumor resection resulted in greater resectability and better outcome. Liver metastasis is considered to be a significant prognostic factor, and may affect outcome. Treatment of liver metastasis may be given priority over that of primary tumors. In this study, liver metastasis was insensitive to chemotherapy when compared with the primary tumor, which supports the strategy proposed by Mentha et al. Okumura et al. reported that liver metastasis was significantly more resistant to 5-FU when compared with the primary tumor in a mouse model [9], [22]. In that study, dihydropyrimidine dehydrogenase (DPD) and thymidylate phosphorylase (TP) mRNA levels increased with repeated liver metastasis. DPD and TP may mediate acquisition of chemoresistance to 5-FU during liver metastasis. Liver metastasis may have a long convalescence from a point of in vitro chemosensitivity.

On the other hand, in the lung metastasis group, chemosensitivity between primary tumors and metastatic tumors was not significantly different. Gorlick et al. reported that thymidylate synthase (TS) mRNA expression was significantly higher in lung metastasis as compared with liver metastasis [23]. However, DPD expression in lung metastasis was low as compared with matched primary tumor, while that of liver metastasis was high [24]. TP, TS, and DPD are actively involved in 5-FU metabolism. DPD-low expression may be related to that lung metastasis is not chemoresistant for primary tumor as compared with liver metastasis.

The present study demonstrated that combination chemotherapy was more than effective than monotherapy. In the liver metastasis group, combination chemotherapy is required because liver metastasis is relatively chemoresistant, and monotherapy is associated with poor outcomes. The CD-DST can assess sensitivity to relatively newer agents. In vitro sensitivity of combination therapy using newer agents can be assessed as well as to combination therapy with 5-FU/SN38 and 5-FU/OHP. The most appropriate anticancer regimen can be identified using the CD-DST analysis. Patients in whom tumors are chemoresistant to conventional chemotherapies according to the CD-DST should be considered for alternative treatment strategies, such as surgery and molecularly targeted drugs [15]–[19]. This would have the benefit of avoiding side effects associated with systemic therapies in patients who would not otherwise benefit from such therapy [18], [19]. In fact, CD-DST might be used to supplement informed consent prior to initiation of therapy.

In summary, the present study demonstrated that in vitro chemosensitivity of liver metastasis was relatively resistant when compared with primary tumors, and showed that combination chemotherapy was more than effective than monotherapy.
